# Design, Manufacture, and Characterization of Auxetic Yarns with Multiple Core/Wrap Structure by Braiding Method

**DOI:** 10.3390/ma15186300

**Published:** 2022-09-10

**Authors:** Sai Liu, Haoyu Chen, Yizhu Li, Zhaoqun Du

**Affiliations:** 1 College of Textile Science and Engineering (International Institute of Silk), Zhejiang Sci-Tech University, Hangzhou 310018, China; 2 Engineering Research Center of Technical Textiles, Ministry of Education, Donghua University, Shanghai 201620, China

**Keywords:** auxetic yarns, tensile properties, Poisson’s ratio, braiding

## Abstract

Auxetic textiles with a negative Poisson’s ratio show significant energy absorption and synclastic curvature characteristics and potential application value in sportsmen protection material. The stability and reliability of the structure and properties of auxetic textiles is also an important factor to assess and promote the application. Thus, auxetic yarns with multiple core/wrap structure were prepared by a 16-spindle braiding machine. It mainly focused on the axial stretching behavior and the relationship between the structure and auxetic effect of yarn samples. The maximum Poisson’s ratio of auxetic yarns was −3.26. The experimental results also showed that the complex yarns still presented an auxetic effect during 30 repeats of cycle stretching. According to the study about the repeatable stretchability and auxetic effect of complex yarns, it could be expected to provide more comfortable, safer, and smarter protective textiles.

## 1. Introduction

Auxetic materials with a negative Poisson’s ratio show the expanding deformation behavior under a stretching load. It presents a wide range of applications in protective clothing, filter materials, biomedical materials, and sensors as a result of special properties in mechanics and structural deformation [1,2,3,4]. A series of studies of textiles [5,6,7], with negative Poisson’s ratios about structure design, preparation methods, mechanical properties, and deformation mechanism have been carried out and reported [8,9,10].

According to the reported experimental results, the first proposed auxetic yarn was with a two-component helical structure [11]. The rigid component with larger modulus was helically wrapped on the surface of the elastic core filament. Under the axial tension, the difference in the mechanical property and the mutual compression effect between the two components also turned the elastic core filament to a bending state from a straight state. With the increasing of the overall contour diameter, Poisson’s ratio of the composite yarn showed a dynamic change trend from positive to negative. Generally, the initial helical wrapping angle, the diameter ratio, and modulus ratio between the two components are considered as the key structural parameters to determine the auxetic effect [12].

Three preparation methods including ring-spinning, hollow spindle, and braiding were proposed to manufacture auxetic yarns in Table 1. Du et al. [13], prepared the auxetic yarn based on the ring-spinning system and the wrap filament was spun to wind around the straight core filament helically. Sloan et al. [14], provided a spinner method of rotation of the feed spool to make the filament wrap on the core filament. Zhang et al. [15], manufactured the auxetic yarn with a bespoke semi-coextrusion system, which was bonding prefabricated core fiber with extruded wrap fiber in a semi-molten state. Ge et al. [8], presented the manufacturing process of two soft yarns and two stiff yarns alternately arranged and fed from the bobbins fixed onto a rotating circular disc and twisted together to form the auxetic plied yarn structure. Jiang et al. [5], proposed an auxetic braided structure based on a helical structural arrangement. Jiang et al. [16], also reported the circular braiding technology to overcome the yarn slippage problem in the conventional helical auxetic yarn structure and a higher magnitude of negative Poisson’s ratio could be achieved.

The excellent energy absorption characteristics and conformability of flexible and elastic negative Poisson’s ratio yarn meet the requirements of durability, protection, and comfort of sports protective materials [17]. However, if we want to promote the application of a negative Poisson’s ratio yarn in different fields, we need to further focus on the stability and effectiveness of the negative Poisson’s ratio effect. The performance, stability, and effectiveness of a negative Poisson’s ratio yarn under repeated tension are closely related to its applicability evaluation. In this paper, the structure and properties of a negative Poisson’s ratio yarn under cyclic stretching mode were studied to find out the failure law of a negative Poisson’s ratio. The morphological changes of complex yarn under constant elongation cyclic stretching were investigated, and the changes of Poisson’s ratio and failure conditions of complex yarn after multiple cyclic stretching were analyzed. The research results of this paper can provide an evaluation basis for the application of negative Poisson’s ratio complex yarn in the field of sports protective materials.

## 2. Materials and Methods

**Figure 1:**
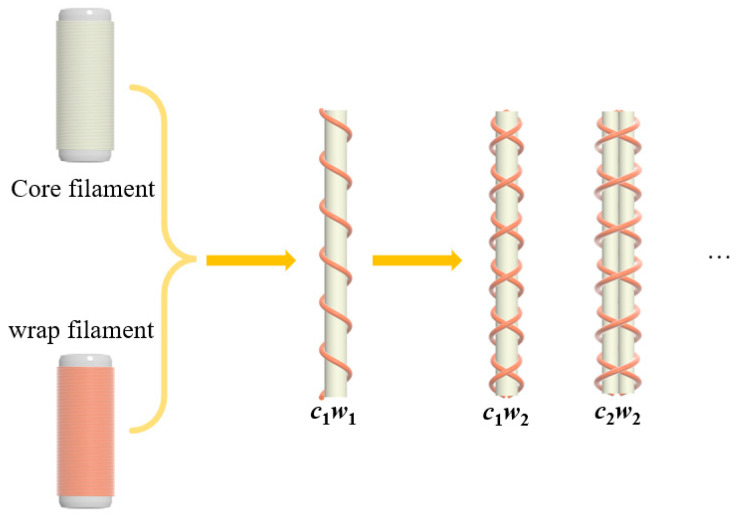
Multiple core/wrap structures of auxetic yarns.

Initial reporting of auxetic yarn was with a helical structure. It was the elastic core filament and the stiff wrap filament that led to the auxetic effect. To improve the stability of the structure and properties, multiple core/wrap structures were provided. As showed in Figure 1, the structure of auxetic yarn was represented by *c*_i_*w*_j_, where c means core filament, w means wrap filament, and i and j mean the number of core and wrap filaments, respectively. The interactive effects between multiple filaments on tensile behavior and properties have been studied.

According to the multiple filaments structure, the braiding method was chose to prepare auxetic yarns. PU (polyurethane, 840D) and Polyamide (120D) were used as core and wrap filament, respectively. PU from the hollow tube of the braiding machine was wrapped by Polyamide filaments unwound from the bobbins. Then, auxetic yarns were prepared to peform axial stretching tests and the contour diameter under axial strain was noted to calculate Poisson’s ratio.

## 3. Results

### 3.1. The Effect of Structure on Mechanical Property

**Figure 2:**
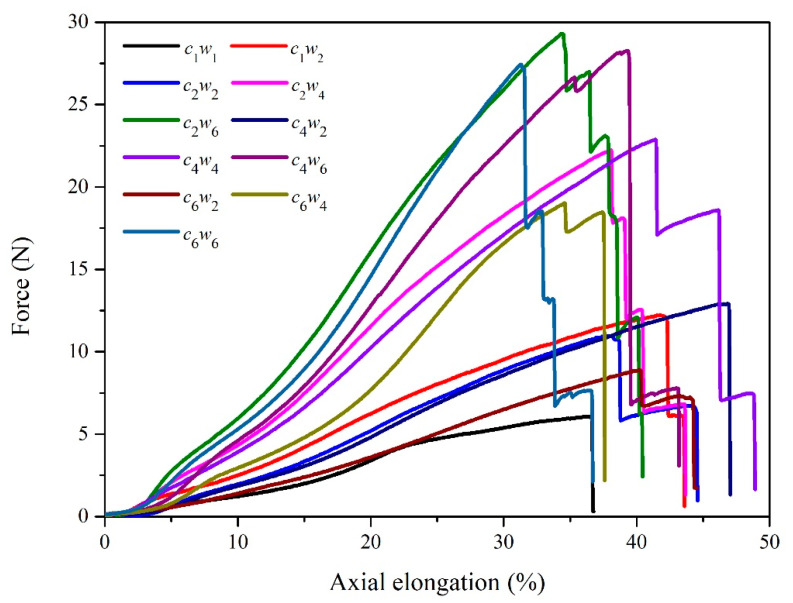
Tensile properties of auxetic yarns.

The structure of yarn is one of the most important parameters that relates to the mechanical properties. As shown in Figure 2, axial stretching behavior of auxetic yarn with different structures was carried out. The breaking elongation of auxetic yarns was from 31.3% to 46.83% and the breaking force was from 6.07° N to 29.29° N. Auxetic yarn with *c*_2_*w*_6_ and *c*_4_*w*_2_ structures presented the highest breaking forces and elongation respectively.

**Figure 3:**
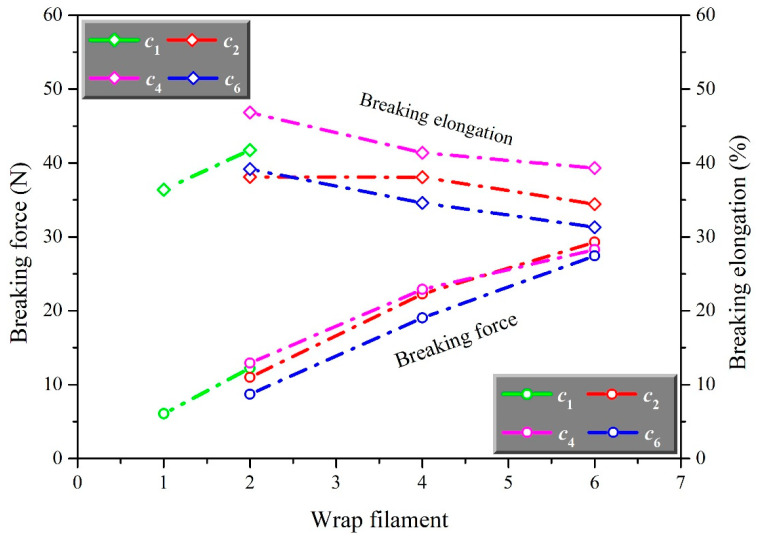
The effect of wrap filament on breaking force and elongation of auxetic yarns.

**Figure 4:**
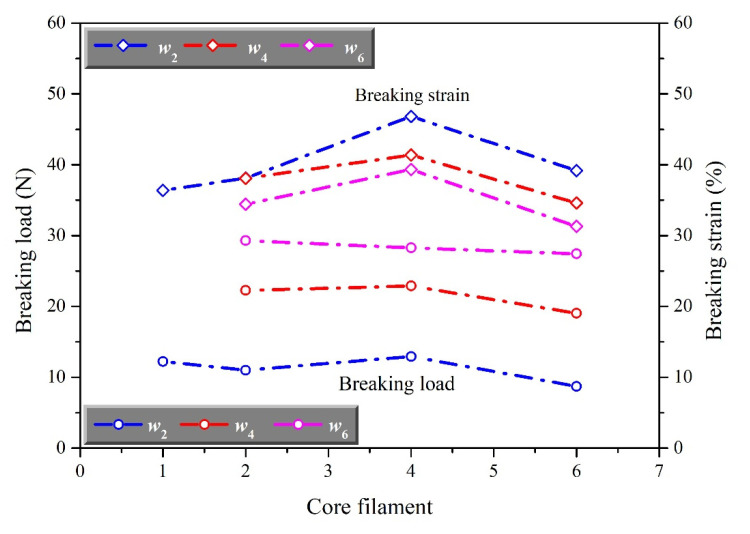
The effect of core filament on breaking force and elongation of auxetic yarns.

In order to further analyze the effect of structure on the mechanical properties of auxetic yarns, the breaking force and elongation of each yarn sample were extracted and studied as shown in Figure 3 and Figure 4.

As shown in Figure 3, auxetic yarn presented a higher breaking force with the increase of the number of wrap filaments. The breaking force of complex yarn with six wrap filaments was much higher than that with one wrap filament. As showed in Figure 4, the breaking elongation of auxetic yarns firstly increased and then decreased with the increase of the number of wrap filaments. The breaking elongation of auxetic yarns with 4 core filaments was much greater than others. However, the breaking force of auxetic yarns with a different number of core filaments showed a closed value. It indicated that the number of core filaments had little effect on the overall breaking force of complex yarns. Compared with the elastic core filaments, the stiff wrap filaments broke first during the stretching process. The key factors of breaking force and breaking elongation of auxetic yarn were the mechanical property of wrap filament and core filament separately. The increasing wrap filaments played a positive role in the auxetic yarns, conversely, limited the axial extension behavior. This was the main reason that auxetic yarns with different structures showed increasing breaking force and similar breaking elongation.

### 3.2. The Effect of Structure on Poisson’s Ratio

**Figure 5:**
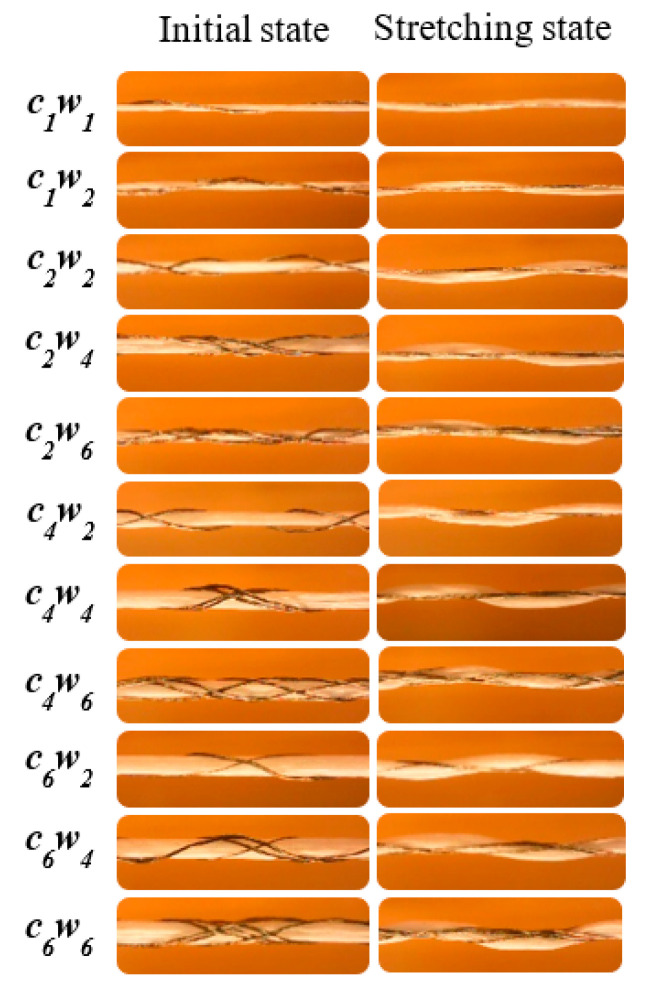
Initial and stretching state of auxetic yarns (scale: 1/3).

Auxetic yarns with multiple core/wrap structures prepared by a braiding machine are shown in Figure 5 including initial state and stretching state. Under 15% axial strain, the helical wrap filaments should have exhibited a straight state. Instead, as a result of the applied force from the wrap components, the core filaments presented bending behavior. The contour diameter of auxetic yarn was bigger than the initial value, which led to a negative Poisson’s ratio.

**Figure 6:**
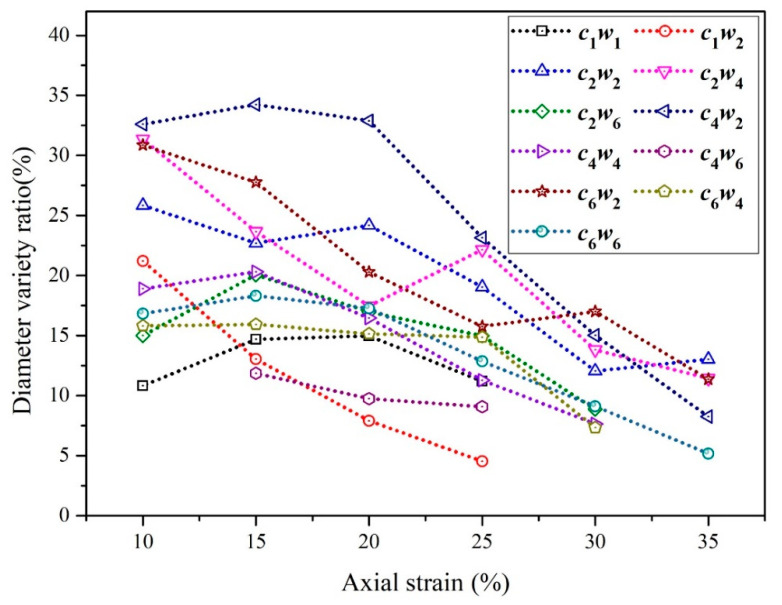
Diameter variety ratio with axial strain of auxetic yarns.

The contour diameters of auxetic yarns under tension were collected and the variety rate based on the initial value is shown in Figure 6. The continuous positive value of the axial strain from 10% to 35% signified the increasing trend of contour diameter of auxetic yarns. During the axial stretching process, the maximum of radial strain of auxetic yarn with *c*_4_*w*_2_ structure was 35% under 10% axial strain.

**Figure 7:**
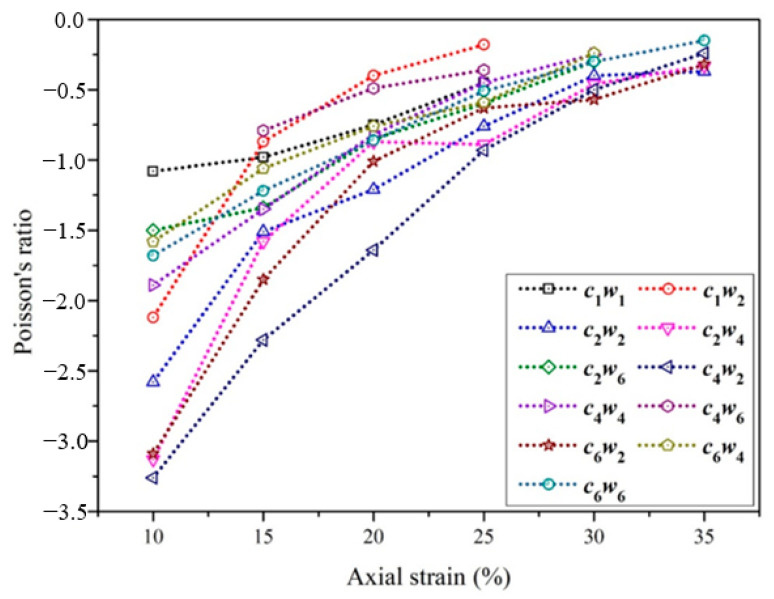
Poisson’s ratio with axial strain of auxetic yarns.

Next, Poisson’s ratio with axial strain was calculated and analyzed. As shown in Figure 7, the maximum Poisson’s ratio of auxetic yarn with *c*_4_*w*_2_ structure was −3.26 under 10% axial strain, which was related to the highest value of contour diameter. With the increase of axial strain, the value of negative Poisson’s ratio gradually decreased, which means the weaker expanding effect of auxetic yarns. The main reason was the state of core filament from straight to completely bending under a compression effect from wrap filaments. Core filament decreased in diameter under axial elongation, which led to the decreasing contour diameter of the auxetic yarn.

**Figure 8:**
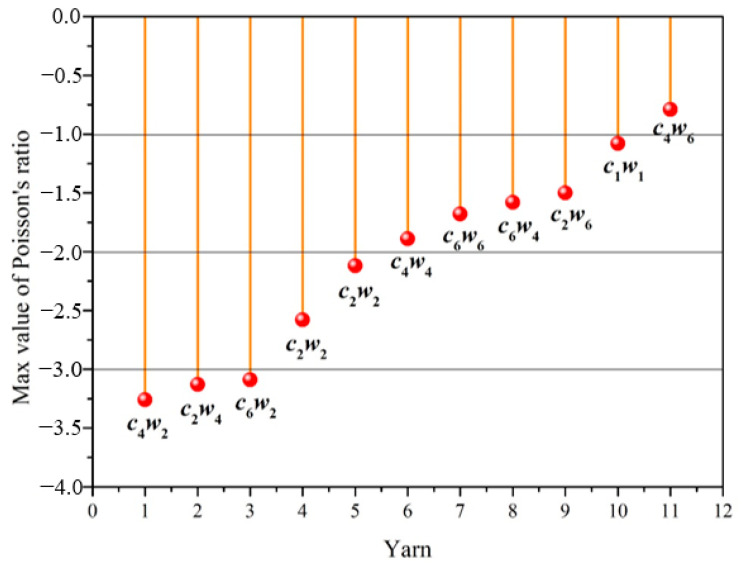
The maximum of negative Poisson’s ratio with axial strain of auxetic yarns.

To analyze the relationship between structure and auxetic behavior easily, the maximum of negative Poisson’s ratio of all auxetic yarns were listed in Figure 8 from largest to smallest. Three yarn samples with *c*_4_*w*_2_, *c*_2_*w*_4,_ and *c*_6_*w*_2_ structures presented a greater auxetic effect. The maximum negative Poisson’s ratios of all three yarns were higher than −3. Compared with the auxetic yarn with two components of c1w1 structure, most yarns with multiple core/wrap structures showed much more significantly expanding behavior. Moreover, when the number of core filaments was four, the auxetic effect of complex yarns decreased with the increasing number of wrap filaments. The auxetic effect of complex yarns with 2 wrap filaments was higher than that of the complex yarns with six wrap filaments. This verified the effect again that the limitation of more wrap filaments to the deformation behavior of core filaments. All of the experimental results noted the auxetic effect of complex yarns with braided structures.

### 3.3. Auxetic Effect under Cycle Stretching

**Figure 9:**
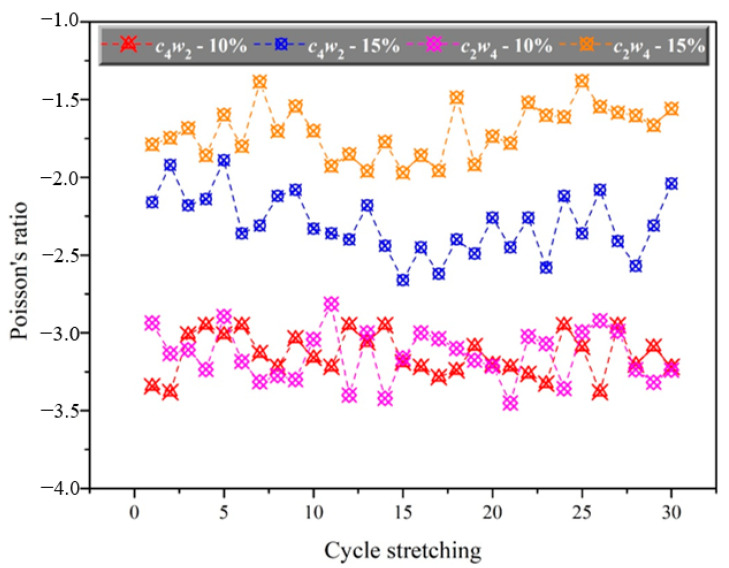
Poisson’s ratio of auxetic yarns under cycle stretching.

In order to study the stability of structure and property, cycle stretching 30 times at 10% and 15% constant elongation of auxetic yarns with *c*_4_*w*_2_ and *c*_2_*w*_4_ structures were carried out. As showed in Figure 9, the corresponding Poisson’s ratio of auxetic yarns with every stretching was provided.

The auxetic effect of both yarns with the constant elongation of 10% was greater than that of yarns with a constant elongation of 15%, which was consistent with Poisson’s ratio curve of auxetic yarns above. The value of Poisson’s ratio stayed negative and varied in a small range during the 30 repeats of cycle stretching. The main reason for the different value may be the structural deformation and test error. However, it also shows the reliability of structure and performance of auxetic yarns and application possibility in the repeated materials.

## 4. Conclusions

Structural deformation and mechanical properties of auxetic yarns with core and wrap filament were studied. The number of core and wrap filaments were helpful to the breaking strain and breaking strength respectively. Under the axial load, the state of the core filament went from straightening to bending. With the increase of the contour diameter the complex yarns showed a negative Poisson’s ratio effect. The maximum negative Poisson’s ratio of the complex yarn was −3.26. Most yarns with multiple core/wrap structures showed much more significant expanding behavior than the yarn with two components of the c1w1 structure. Moreover, both yarns with *c*_4_*w*_2_ and *c*_2_*w*_4_ structures presented an auxetic effect under cycle stretching 30 times at 10% and 15% constant elongation. It is expected to lay a foundation for the further optimizing of the properties and promoting the application of auxetic yarns.

## Figures and Tables

**Table 1:** Preparation methods of auxetic yarns.

Preparation Method	Materials	Structure	Parameters	Maximum of Negative Poisson’s Ratio
Ring-spinning	Core: rubber filament Wrap: nylon filament	Helical Wrapping structure	Initial helical angle: 25°~35°	>−2
Hollow spindle	Core: polyurethane Wrap: nylon filament	Helical Wrapping structure	Initial helical angle: 13°~25°	>−2
Braiding	Stiff yarn: polyester Core/Elastic yarn: polyester and rubber	Tubular braided structure	Initial Braiding angle: 12°~36°	>−5

## Data Availability

Not applicable.
